# Radiological risk factors in patellar instability: a comparative analysis of single-episode, recurrent, and habitual patella dislocation

**DOI:** 10.1186/s43019-025-00292-3

**Published:** 2025-10-01

**Authors:** Devendra K. Chouhan, Prasoon Kumar, Vishnu Baburaj, Pratik M. Rathod, Supreeth Kumar, Mahesh Prakash

**Affiliations:** 1 Department of Orthopaedics, PGIMER, Chandigarh, India; 2 PGIMER, Chandigarh, India

**Keywords:** Patellar instability, Trochlear dysplasia, TT–TG distance, CD ratio, Radiological risk factors, Habitual dislocation, Recurrent dislocation., *Level of evidence* III (retrospective cohort study)

## Abstract

**Background:**

Patellar instability is a multifactorial condition with varying severity, categorized into single-episode, recurrent, and habitual dislocations. This study aims to assess the association and the strength of association between clinical subtypes of patellofemoral instability (PFI) (single-episode, recurrent, and habitual patellar dislocation) and the frequency, severity, and cumulative presence of three key radiological risk factors: trochlear dysplasia, tibial tubercle–trochlear groove (TT–TG) distance, and the Caton–Deschamps (CD) ratio.

**Methods:**

This was a retrospective observational study conducted from January 2018 to December 2024 on 106 patients reported in the outpatient department (OPD) with various type of patellar instability (124 knees; 39 knee SPD, 73 knee RPD, and 12 knee HPD). Three radiological parameters (trochlear dysplasia, tibial tuberosity–trochlear groove (TT–TG) distance with > 15 mm defined as high), and Caton–Deschamps (CD) ratio (> 1.2 indicating patella alta) were evaluated using magnetic resonance imaging (MRI). Chi-squared test and Cramér’s *V* statistical methods were applied for analyzing the strength of association.

**Results:**

The study identified prevalence of trochlear dysplasia in 63.7%, high TT–TG distance in 32.2%, and patella alta in 45.2% of knees with varying types of patellar instability. Trochlear dysplasia showed the strongest association, particularly with habitual dislocations (100%). Prevalence of high TT–TG progressively increased from single-episode (17.9%) to recurrent (32.9%) to habitual patella dislocation (75%). In contrast to trochlear dysplasia and high TT–TG distance, patella alta was found to have a weaker association in characterizing the patellar instability. Notable, correlation was observed with severity of patellar instability and the number of risk factors, with cases with habitual patella dislocation most frequently showing multiple contributing factors.

**Conclusions:**

Our study found that patients with HPD, the most severe clinical form of patellar instability, had pronounced trochlear dysplasia, higher TT–TG distance, and a greater number of radiological risk factors, followed by RPD and finally SPD. Among the parameters, trochlear dysplasia and TT–TG distance showed stronger association with clinical types, while patella alta had a weaker correlation.

## Background

Patellar instability constitutes a multifaceted and intricate condition that significantly impacts the biomechanical functions of the knee joint, frequently resulting in pain, instability, and enduring functional limitations. The clinical manifestations of patellar instability in affected individuals encompass a spectrum ranging from single episode of patella dislocation to recurrent and habitual dislocations [1]. Each of these categories displays unique clinical presentations and characteristics, wherein a solitary episode of patellar dislocation typically involves a noteworthy history of trauma, in contrast to individuals experiencing recurrent patellar instability [2]. Conversely, habitual patellar dislocation often manifests at a very early age, occurring with each instance of knee flexion, which serves as a contrasting characteristic when compared with the other classifications where patellar instability is predominantly observed during extension [1].

Despite the variability in clinical manifestations, all forms of patellar instability exhibit common radiological features, including trochlear dysplasia, an increased tibial tuberosity–trochlear groove (TT–TG) distance, and patella alta, among others. Trochlear dysplasia, in particular, has been recognized as the most prevalent structural anomaly correlated with recurrent and habitual dislocations [3]. An elevated TT–TG distance signifies abnormal lateralization of the tibial tuberosity, generating a substantial lateral force vector on the patella and thereby contributing to patellar instability [4, 5], while an elevated CD ratio (patella alta) may further aggravate patellar instability owing to the delayed engagement of the patella within the trochlear groove [6].

These radiological risk factors may occur in isolation or in various combinations, as delineated in the new classification system proposed by Frosch et al.[7]. Each factor independently necessitates a tailored management strategy and serves as a prognostic indicator [8]. Therefore, we hypothesize that the clinical severity of patellofemoral instability (PFI) correlates with the cumulative radiological burden.

This study aims to assess the association—and the strength of that association—between clinical subtypes of PFI (single-episode, recurrent, and habitual patellar dislocation) and the frequency, severity, and cumulative presence of three key radiological risk factors: trochlear dysplasia, tibial tubercle–trochlear groove (TT–TG) distance, and the Caton–Deschamps (CD) ratio.

## Materials and methods

This was a retrospective observation study conducted in Postgraduate Institute of Medical Education and Research (PGIMER, Chandigarh) after obtaining appropriate ethical approval. Demographics (age, gender, and affected side), clinical data (number of time patella dislocated), and radiological data (MRI) records of all patients who presented with patella dislocation during the period from January 2018 to December 2024 were collected. Any case with history of previous knee surgery, lack of required minimum clinicoradiologiocal details, and SDP that underwent patellar stabilization procedure were excluded from the study. As per hospital protocol, MRI of the knee was done in full extension using a knee coil in a 3 T/1.5 T Siemens Magnetom Aera. The dataset used was anonymized to maintain patient confidentiality. Ethical approval was obtained for the secondary use of the data for analysis.

On the basis of the history about the number of times the patella dislocated, each patient was classified as having single-episode patella dislocation (SPD), recurrent patella dislocation (RPD), or habitual patella dislocation (HPD) [9]. Radiological records were viewed using a Radiant Dicom viewer (version 2024.1), and measurement of primary radiological risk factors was done following a predefined methodology.

### Radiological risk factors

**Fig. 1:**
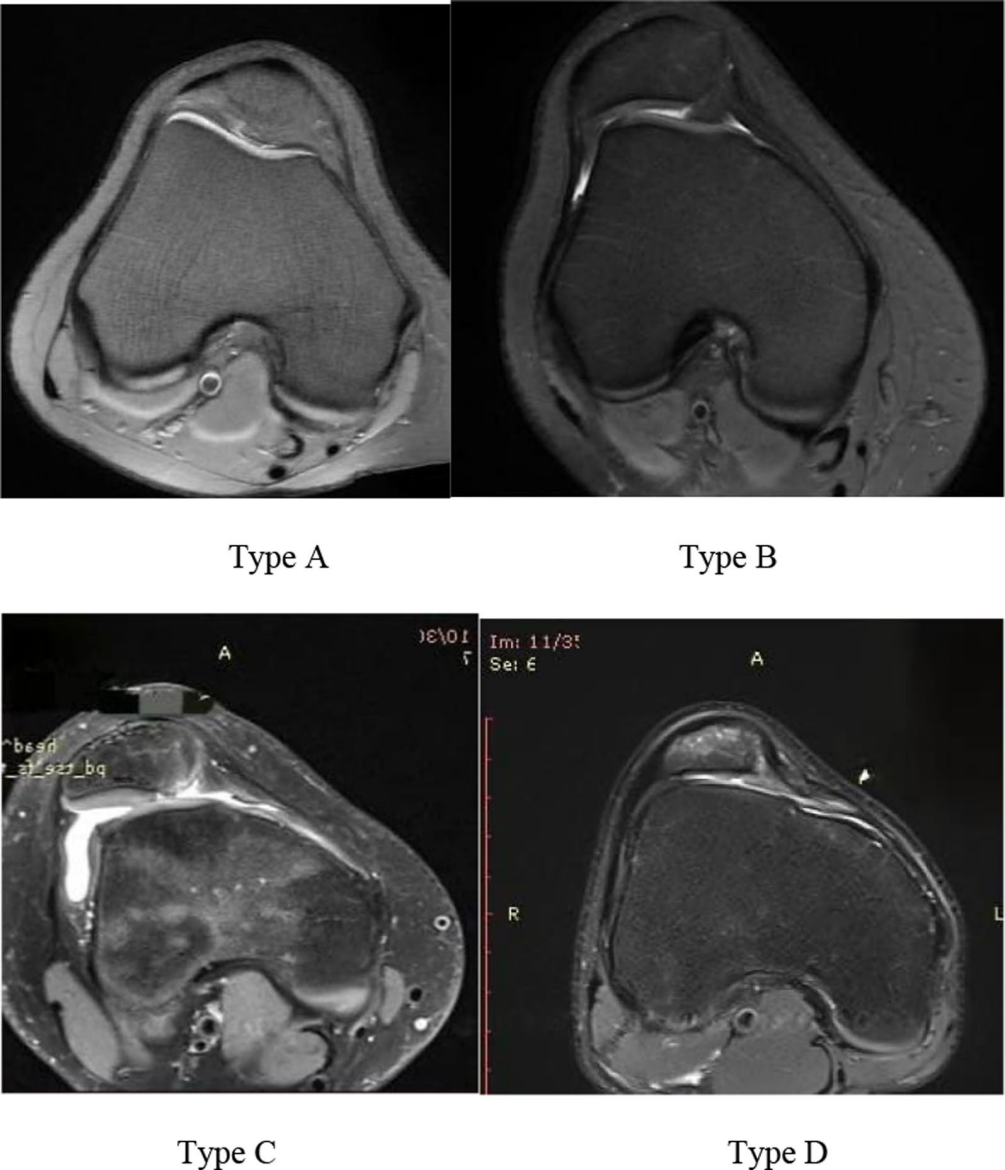
Various types of trochlear dysplasia shown on axial MRI

**Fig. 2:**
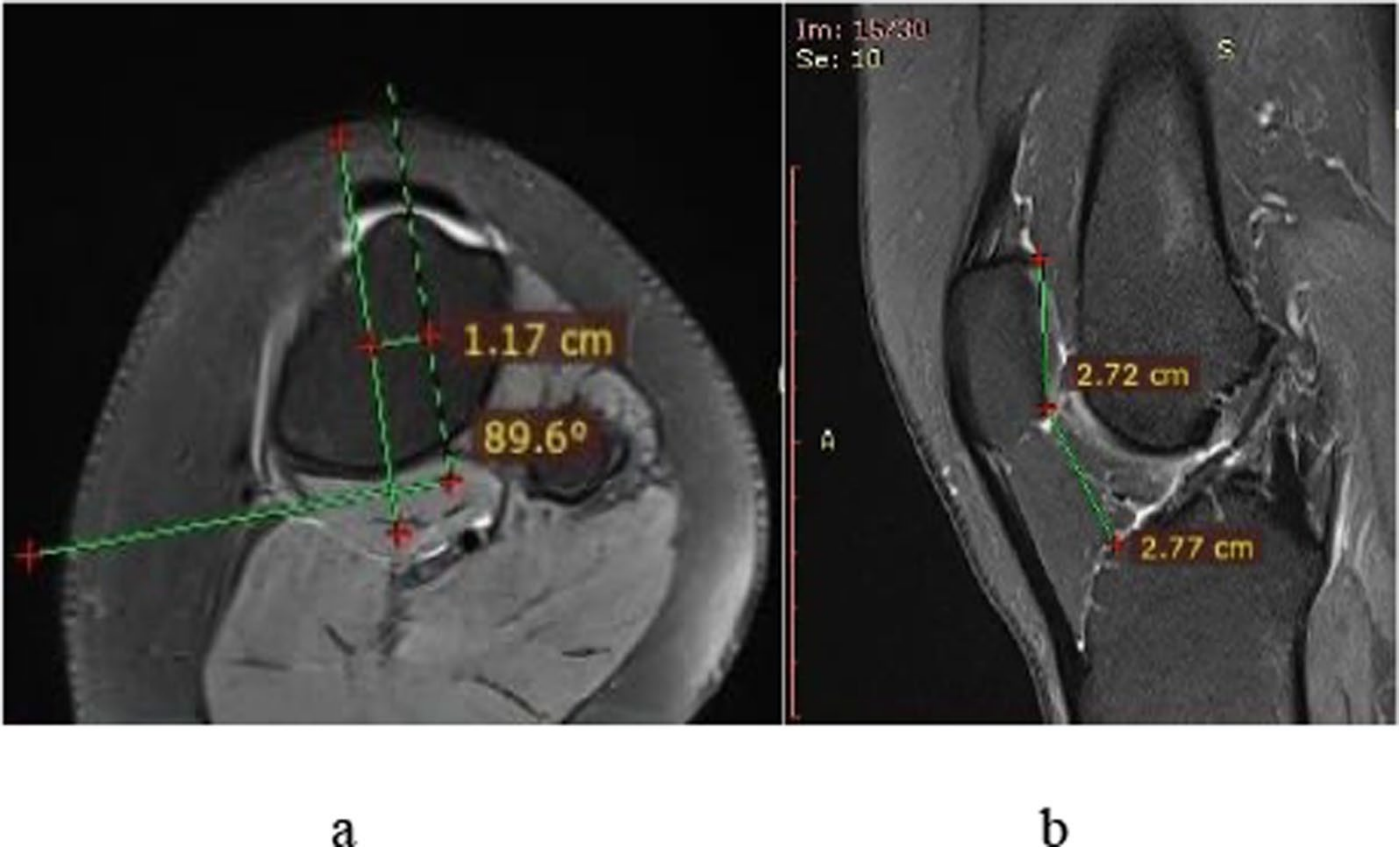
The method of measuring TT–TG (a) and CDI (b)

- Trochlear dysplasia was classified using Dejour’s classification [9, 10] as type A, B, C, and D, and trochlear depth was measured on the most proximal axial MRI section showing the entire cartilaginous trochlea [3, 8]:
  o Type A represents fairly shallow trochlea (< 3 mm trochlear depth);
  o Type B is characterized by flat or convex trochlea;
  o Type C shows convex lateral facet and a hypoplastic medial condyle;
  o Type D involves asymmetry of trochlear facets plus vertical join or cliff pattern (Fig. 1).
- TT–TG distance was measured using Pfirmann’s method [11]. The first craniocaudal image showing complete cartilaginous trochlea was selected to determine the deepest point of trochlea. A line perpendicular to the posterior condylar axis was drawn, passing through the deepest point of trochlea. These lines were copied onto the most proximal image showing complete attachment of patellar tendon to tibial tuberosity. Another perpendicular line was drawn, passing through the center of patellar tendon insertion. TT–TG was measured as the distance between these two perpendicular lines in millimeters (Fig. 2). A distance of > 15 mm was considered as high and ≤ 15 mm as normal [5].
- The Caton–Deschamps (CD) ratio was measured on sagittal cuts of MRI as the ratio of the shortest line between the caudal margin of patellar articular surface and the anterior aspect of the tibial plateau and the greatest length of the patellar articular surface [5](Fig. 2). A CD ratio of ≥ 1.2 was considered as high (patella alta) [4], whereas a CD ratio < 0.8 is called patella baja. A CD ratio of 0.8–1.2 is regarded as normal [12].

### Statistical analysis

All analyses were performed using SPSS version 24.1. For continuous variables such as age, the mean and range were calculated, while categorical variables—including gender, presence of trochlear dysplasia, elevated TT–TG distance, and CD ratio—were analyzed using prevalence rates and proportions. For the purpose of statistical evaluation, Dejour types B, C, and D were collectively categorized as high-grade trochlear dysplasia. To assess associations between categorical variables, the chi-squared test was utilized. The strength of these associations was measured using Cramér’s *V* coefficient, with values interpreted as weak (≥ 0.1), moderate (≥ 0.3), or strong (≥ 0.5) based on Cohen’s guidelines [13]. To analyze the association between the number of radiological risk factors and type of instability, SPD, RPD, and HPD were labeled as mild, moderate, and severe form of patellar instability. The chi-squared test was used to test for the association, and Cramér’s *V* was used for the strength of association. To achieve a power of 80% at a significance level of 0.05 with a moderate effect size (Cramér’s *V* = 0.3) for a chi-squared test with three groups (SPD, RPD, and HPD), the required total sample size was approximately 107 with 35–40 in each group.

## Results

In the present investigation, we conducted a comprehensive analysis of the radiological anatomical risk factors that contribute to patellofemoral instability in a cohort of 106 patients (124 knees), emphasizing trochlear dysplasia, TT–TG distance, and the CD ratio across three types of patellar instability: single dislocation (SPD; 39 knees), recurrent dislocation (RPD; 73), and habitual dislocation (HPD; 12) (Tables 1 and 2). In view of the unequal sample size in the groups, we carried out post hoc power analysis to evaluate the adequacy of sample size. The results indicated that the study was sufficiently powered to detect moderate associations of trochlear dysplasia and TT–TG distance (power > 0.80) in characterizing the type of patellar instability. However, the power for detecting differences in patella alta was lower (power of 0.51) (Table 3).

**Table 1:** Results of radiological parameters among various types of instability

Type of patellar instability	Age (mean, range)	Gender ratio (F:M)	Side	Type of trochlear dysplasia	TT–TG distance (high, normal)	CD ratio (high, normal)	Number of radiological risk factors
Habitual (*n* = 12)	20.9 [15.0–43.0]	1.2	Lt -5 Rt – 5 B/L- 1	A: 0, B: 4, C: 7, D: 1	High: 9, normal: 3	High: 4, normal: 6 low:1	0: 0, 1: 0, 2: 11, 3: 1
Recurrent (*n* = 73)	23.1 [14.0–42.0]	1.76	Lt: 21 Rt: 20 B/L: 15 + 2*	A: 13, B: 21, C: 10, D: 4	High: 24, normal: 49	High: 32, normal: 40	0: 11, 1: 31, 2: 20, 3: 11
Single episode (n = 39)	19.9 [13.0–33.0]	0.81	Lt: 26 Rt: 11 B/L: 2*	A: 6, B: 10, C: 3, D: 0	High: 7, normal: 32	High: 20, normal: 19	0: 10, 1: 15, 2: 11, 3: 3
*p*-Value	0.053	0.183	0.016	*p* = 0.003	*p* = 0.001	0.024	0.001
Cramér’s *V*, ≥ 0.1 weak ≥ 0.3 moderate ≥ 0.5 strong			0.27	0.35	0.33	0.24	0.3

^*^Two patients who had bilateral patellar instability had recurrent dislocation on one side and single-time dislocation of patella on the other side

**Table 2:** Radiological risk factors in various types of patellar instability

Parameter	Habitual	Recurrent	Single episode
Sulcus depth	Flat to convex	3 ± 2.5 mm	5 ± 3.5 mm
CD ratio	1.25 ± 0.3	1.32 ± 0.18	1.35 ± 0.28
TT–TG distance	19.37 ± 8.52 mm	13.81 ± 2.64 mm	11.69 ± 3.6 mm

**Table 3:** Post hoc analysis for computation of study power

Parameter	Cramér’s *V*	Estimated power
Trochlear dysplasia	0.35	0.871
High TT–TG	0.33	0.823
Patella alta	0.24	0.509

The average age of the participants was 21.76 years (range 13–43 years). No statistically significant variation in age was discerned among the various categories of patellar instability. The sample comprised 59 females (55.66%) and 47 males (44.33%). The gender distribution pattern was consistent in cases of RPD and HPD, while SPD exhibited a comparatively higher prevalence among male patients. Nonetheless, no significant correlation was identified between the type of instability and gender. The left knee was affected in 52 patients, which was substantially greater than the 36 patients with right knee involvement (*p* = 0.016). Bilateral involvement was significantly more prevalent in the RPD group compared with the SPD or HPD groups, with *p*-values of 0.016 and 0.496, respectively.

### Radiological risk factor analysis

Trochlear dysplasia was identified in 63.7% of the knees and exhibited significant variation in relation to the type of patellar instability (*p* = 0.003), indicating a moderate strength of association. Within the SPD cohort, 20 out of 39 knees (51.3%) displayed normal trochlear morphology, whereas all patients with HPD presented with high-grade trochlear dysplasia (100%). Of the RPD group, 65.7% showed trochlear dysplasia. Thje trochlear was observed as flat to convex in all cases with HPD, and the trochlear depth in RPD and SPD was 3 ± 2.5 mm and 5 ± 3.5 mm, respectively.

High TT–TG distance was observed in 32.2% of the knees, and this distance exhibited significant variation with respect to the type of patellar instability (*p* = 0.001), demonstrating a moderate strength of association. A substantial proportion of the HPD group (75%) and RPD group (32.9%) exhibited elevated TT–TG distances, underscoring a noteworthy relationship between this parameter and patellar instability. The TT–TG distance in HPD, RPD, and SPD was 19.37 ± 8.52 mm, 13.81 ± 2.64 mm, and 11.69 ± 3.6 mm, respectively.

The CD ratio was elevated in 45.2% of the examined knees, displaying significant variation according to the type of patellar instability (*p* = 0.024). However, a weak strength of association was noted. RPD and SPD had comparable CD ratios (1.32 ± 0.18 and 1.35 ± 0.28, respectively), while habitual dislocations showed a slightly lower average CD ratio (1.25 ± 0.3) (Table 2). Remarkably, only the HPD group presented cases with patella baja (8.3%).

### Frequency of multiple radiological risk factors

Among the knees with HPD, 11 (91.7%) exhibited two radiological risk factors, while 1 knee (8.3%) presented all three radiological risk factors. In the RPD group, 11 knees (15.0%) displayed three radiological risk factors, 20 (27.4%) exhibited two radiological risk factors, 31 (42.4%) had one radiological risk factor, and 11 (15.0%) were identified as having no radiological risk factors. Among the SPD cohort, three knees (7.7%) presented three radiological risk factors, two of which had RPD on the contralateral side. Additionally, 11 knees (28.2%) with SPD exhibited two radiological risk factors, 15 (38.5%) had one radiological risk factor, and 10 (25.6%) presented no radiological risk factors. Statistical analysis revealed a significant association between the increasing severity of patellar instability and escalating number of radiological risk factors (*p* = 0.001), corroborated by a moderate strength of association (Table 4).

**Table 4:** Frequency of radiological risk factors in various types of patellar instability

Type of instability	Trochlear dysplasia					High TT–TG	Patella alta
	Type A	Type B	Type C	Type D	Total		
Habitual	0 (0%)	4 (33.3%)	7 (58.3%)	1 (8.3%)	12 (100%)	9 (75%)	4 (33.3%)
Recurrent	13 (17.8%)	21 (28.7%)	10 (13.7)	4 (5.5%)	48 (65.7%)	24 (32.9%)	32 (43.8%)
Single episode	6 (15.4%)	10 (25.6%)	3 (7.7%)	0 (0%)	19 (48.7%)	7 (17.9%)	20 (51.3%)
Total prevalence	15.3%	28.2%	16.1%	4.0%	63.7%	32.2%	45.2%

## Discussion

The observations in this study elucidate pathoanatomical distinctions concerning the radiological prevalence of trochlear dysplasia, elevated TT–TG difference, and patella alta in a cohort of cases with various types of patellar instability. Trochlear dysplasia was identified in 63.7% of the subjects, a significantly elevated TT–TG distance was present in 32.2%, and patella alta was noted in 45.2% across the various types of patellar instability. On reviewing literature, most studies were found to focused on cases with recurrent patellar instability, but none have been so comprehensive by including all three types of patellar instability (Table 5).

**Table 5:** Literature on imaging-based risk factors in various types of patellar instability

Study	Types of instability addressed			Radiological risk factors addressed		
	SPD	RPD	HPD	TD	TT–TG	Patella alta
Kohlitz [3]	✓	✓	✗	✓	✓	✓
Lin Huang [14]	✗	✓	✓	✓	✓	✓
H. Dejour [15]	✓	✓	✗	✓	✓	✓
Balcarek [8]	✓	✓	✗	✓	✓	✓
Robert Steensen [16]	✗	✓	✗	✓	✓	✓
Askenberger [5]	✓	✗	✗	✓	✓	✓
P. Megremis [17]	✗	✓	✗	✓	✓	✓
Friedman [18]	✓	✗	✗	✓	✓	✗
Niclas Eysturoy [19]	✓	✗	✗	✓	✗	✗

However, trochlear dysplasia was recognized as the predominant radiological risk factor across all forms of patellar instability and was observed with increasing frequency concomitant to the clinical severity. Notably, all participants with HPD manifested high-grade trochlear dysplasia (100%), in contrast to 47.9% of knees classified under RPD and 33.3% within SPD cases. High-grade trochlear dysplasia was markedly more pronounced in HPD and RPD, which is consistent with observations documented in various studies [14, 20, 21].

Moreover, beside the severity of trochlear dysplasia, the prevalence of high TT–TG distance exhibited variability among the disparate forms of patellar instability, with proportions of 17.9% in SPD, 32.9% in RPD, and 75% in HPD, thereby indicating a progressive increase in prevalence of TT–TG distance across these types. The substantial correlation between increased TT–TG distance and recurrent instability aligns with findings articulated by Balcarek et al. [8], Christensen et al. [22], and Sanders et al. [23]. Nevertheless, corroborating our study, observations by Sillanpää et al. [24] and Wierer et al. [25] identified a comparatively weaker association of patella alta in characterizing the patellar instability, which stands in contrast to findings by Sundararajan et al. [21], Christensen et al. [22], and Lin Huang et al. [14]. It is imperative to underscore that our study cohort was notably heterogeneous, encompassing 11 patients (12 knees) with habitual patellar dislocation, among which only 4 knees demonstrated patella alta, while 1 knee displayed patella baja. Such findings in patients with HPD corroborate observations made by Zhang et al. [20]. The reduced prevalence of patella alta (33.3%) within the HPD group, compared with 43.8% in RPD and 51.3. % in SPD, suggests potential differences in pathoanatomy as a plausible inciting factor [20].

Additionally, strong correlation was observed between severity of patellar instability and an increasing number of radiological risk factors, which corroborates findings reported by Lin Huang et al. [14], Osman et al. [26], Lewallen et al. [27], and Hiemstra et al. [28]. The lower incidence of various radiological anatomical risk factors in patients with SPD substantiates the classification proposed by Hiemstra et al. [28] and supports the assertion that not all SPD cases will ultimately result in recurrent patellar instability. In our analysis, out of a total of 39 patients with SPD, 35.90% were determined to possess one radiological risk factor, 7.69% exhibited two risk factors, and two patients presented with three risk factors. These observations are recognized as instrumental in prognosticating the likelihood of recurrence [16, 26, 27, 29]. Furthermore, two patients exhibited RPD on the contralateral side, which itself constitutes an additional risk factor, and both of these patients possessed all three anatomical risk factors, thereby validating bilateral disease as a clinical risk factor for a heightened degree of patellar instability [30, 31].

Our research findings were contextualized within the framework of extant literature spanning multiple continents. In geographic locales such as Europe, empirical studies demonstrate a substantial prevalence of severe trochlear dysplasia among individuals experiencing patellar instability [3, 5, 8, 15, 24, 25, 32], with reported rates escalating to as high as 85%. In contrast, investigations conducted in Asia [21, 33] reveal comparatively lower frequencies, implying that either genetic predispositions or environmental variables may significantly affect the anatomical vulnerabilities associated with patellar instability. The TT–TG distance similarly exhibits analogous patterns, with European cohorts presenting elevated abnormal values relative to their counterparts in Asia and Africa [26]. This observed variability accentuates the critical necessity for regional considerations in the diagnosis and management of patellar instability. Upon evaluating the CD ratio, our findings suggest a more uniform prevalence across different continents, although certain studies and our observations indicate that its correlation with instability may be less robust compared with that of trochlear dysplasia and TT–TG distance [24, 25].

## Limitations

The nature of the study was retrospective, thus precluding our ability to infer causal relationships with onset, activity level, and various other secondary regional (valgus knee alignment and torsional studies of lower limb) and systemic factors, etc. However, it is noteworthy that this study aimed to look at primary risk factor as proposed by Dejour et al. Second, interobserver and intraobserver variability were not assessed in the measurement of radiological parameters, which may affect the consistency and reproducibility of the findings. However, all the measurements were done on the basis of consensus between an orthopedic surgeon with 19 years of experience in the field and a radiologist with more than 20 years of experience in musculoskeletal radiology. Third, the unequal distribution of cases among the study groups resulted in an underrepresentation of high-grade patellar dysplasia (HPD) and an overrepresentation of recurrent patellar dislocation (RPD). In spite of this unequal distribution of cases in each group, on post hoc analysis, the study power was moderate. Nevertheless, the prevalence of HPD is inherently lower within the general population when juxtaposed with RPD [14, 34], a trend that is similarly reflected in the investigation conducted by Zhang et al. [20].

## Conclusions

Our study demonstrated that patients with HPD, representing the most clinically severe form of patellar instability, also exhibit the most severe form of trochlear dysplasia and higher TT–TG distance, compounded by a greater number of radiological risk factors. Radiological severity was relatively less in cases with RPD, and least in patients with SPD. Among the three studied radiological parameters, trochlear dysplasia and TT–TG distance exhibited the strongest association in characterization of the clinical types of patellar instability, whereas patella alta showed a comparatively weaker strength of association.

## Data Availability

The datasets used and/or analyzed during the current study are available from the corresponding author on reasonable request.

## Abbreviations

PFI: Patellofemoral instability
CD: Caton–Deschamps
HPD: Habitual patella dislocation
MRI: Magnetic resonance imaging
OPD: Outpatient department
RPD: Recurrent patella dislocation
SPD: Single-episode patella dislocation
TT–TG: Tibial tuberosity trochlear groove
